# Neuromelanin related ultra-high field signal intensity of the locus coeruleus differs between Parkinson’s disease and controls

**DOI:** 10.1016/j.nicl.2023.103479

**Published:** 2023-07-22

**Authors:** Amée F. Wolters, Margot Heijmans, Nikos Priovoulos, Heidi I.L. Jacobs, Alida A. Postma, Yasin Temel, Mark L. Kuijf, Stijn Michielse

**Affiliations:** a Department of Neurology, Maastricht University Medical Center, Maastricht, The Netherlands; b School for Mental Health and Neuroscience, Maastricht University, Maastricht, The Netherlands; c Spinoza Centre for Neuroimaging, Amsterdam, The Netherlands; d Computational Cognitive Neuroscience and Neuroimaging, Netherlands Institute for Neuroscience, Amsterdam, The Netherlands; e Department of Cognitive Neuroscience, Faculty of Psychology and Neuroscience, Maastricht University, Maastricht, The Netherlands; f Gordon Center for Medical Imaging, Department of Radiology, Massachusetts General Hospital, Harvard Medical School, Boston, USA; g Department of Radiology and Nuclear Medicine, Maastricht University Medical Centre, The Netherlands; h Department of Neurosurgery, Maastricht University Medical Center, Maastricht, The Netherlands

**Keywords:** Parkinson’s disease, Magnetic resonance imaging, Ultra-high field, Neuromelanin, Locus coeruleus, Substantia nigra

## Abstract

**Introduction:**

Neuromelanin related signal changes in catecholaminergic nuclei are considered as a promising MRI biomarker in Parkinson’s disease (PD). Until now, most studies have investigated the substantia nigra (SN), while signal changes might be more prominent in the locus coeruleus (LC). Ultra-high field MRI improves the visualisation of these small brainstem regions and might support the development of imaging biomarkers in PD.

**Objectives:**

To compare signal intensity of the SN and LC on Magnetization Transfer MRI between PD patients and healthy controls (HC) and to explore its association with cognitive performance in PD.

**Methods:**

This study was conducted using data from the TRACK-PD study, a longitudinal 7T MRI study. A total of 78 early-stage PD patients and 36 HC were included. A mask for the SN and LC was automatically segmented and manually corrected. Neuromelanin related signal intensity of the SN and LC was compared between PD and HC.

**Results:**

PD participants showed a lower contrast-to-noise ratio (CNR) in the right SN (p = 0.029) and left LC (p = 0.027). After adding age as a confounder, the CNR of the right SN did not significantly differ anymore between PD and HC (p = 0.055). Additionally, a significant positive correlation was found between the SN CNR and memory function.

**Discussion:**

This study confirms that neuromelanin related signal intensity of the LC differs between early-stage PD patients and HC. No significant difference was found in the SN. This supports the theory of bottom-up disease progression in PD. Furthermore, loss of SN integrity might influence working memory or learning capabilities in PD patients.

## Highlights

• Neuromelanin related signal intensity of the LC differs between early-stage PD and HC.
• Neuromelanin related signal intensity of the SN did not differ between groups.
• These results support the theory of bottom-up disease progression in PD.
• Loss of SN integrity might influence working memory or learning capabilities in PD.

## 1 Introduction

Parkinson’s disease (PD) is the second most common neurodegenerative disorder after Alzheimer’s disease and its incidence is expected to grow exponentially in the coming years (Dorsey et al., 2018). It is characterized by motor symptoms, such as bradykinesia, rigidity and tremor, and non-motor symptoms, such as depression, cognitive impairment and autonomic dysfunction (Jankovic, 2008, Postuma et al., 2015). The diagnosis of PD is based on clinical observations and therefore remains challenging. Patients often experience a delay in diagnosis and about a quarter of the clinical diagnoses of parkinsonism are incorrect (Joutsa et al., 2014).

In clinical practice, Magnetic Resonance Imaging (MRI) is often used as a method to exclude other potential causes of parkinsonian symptoms. To date, it is impossible to diagnose a patient with PD solely based on MRI characteristics. However, in recent years a lot of effort has been put into the development of MRI techniques that could be of interest in search for a potential PD biomarker (Lehericy et al., 2017). Magnetization Transfer (MT) MRI, a magnetization exchange between short T_2_ water protons bound to macromolecules and freely moving intra- and extra-cellular protons, has been shown to measure neuromelanin related signal in small catecholaminergic nuclei (Priovoulos et al., 2018). Moreover, ultra-high-field 7T MRI provides an increased spatial resolution and a higher signal-to-noise (SNR) ratio, thereby enabling the visualisation of these small brainstem nuclei (Prange et al., 2019). This new imaging techniques and higher field strength are therefore of specific interest in the diagnostic work-up of PD.

The visualisation of neuromelanin related signal on anatomical MRI is considered one of the most promising radiological biomarkers in PD. The insoluble pigment neuromelanin is a by-product of the oxidative metabolism of dopamine and noradrenaline and is predominantly situated in the substantia nigra (SN) and locus coeruleus (LC) (Prange et al., 2019, Rispoli et al., 2018). Although the biological source of the neuromelanin contrast on neuromelanin sensitive MRI sequences is not entirely clear, it is most likely indirectly related to neuromelanin cell density (Watanabe et al., 2019, Priovoulos et al., 2020). In PD, MRI studies consistently show lower neuromelanin related signal intensity and volume in the SN compared to healthy controls (HC) (Castellanos et al., 2015, Schwarz et al., 2011, Reimão et al., 2015). Longitudinal changes in SN signal intensity on MRI might even be able to serve as a biomarker for disease progression (Matsuura et al., 2016, Pavese and Tai, 2018). However, until now this has mainly been investigated with 3T MRI, while it might be more reliable to visualize these small nuclei with 7T MRI (Prange et al., 2019). Furthermore, the vast majority of 3T MRI studies have focussed on the SN, while previous research suggests that in most PD patients neuromelanin related signal alterations may be more prominent in the LC (Isaias et al., 2016). This is in line with the progression of neuropathology according to the Braak hypothesis, which states that the propagation of pathology goes via the brainstem upwards (Braak et al., 2002). However, recent evidence suggests two different spreading routes for the pathologic process in PD, which could explain why most, but not all patients display more prominent alterations in the LC (Borghammer et al., 2022).

Furthermore, noradrenergic neurons in the LC are important for cognitive performance (Mather and Harley, 2016). This is underlined by the fact that LC alterations have extensively been reported in Alzheimer’s disease (Theofilas et al., 2017). Moreover, in a previous 3T MRI study a lower signal in the LC was found in PD patients with cognitive impairment compared to HC participants (Li et al., 2019). It is therefore suggested that alterations in LC functioning might contribute to cognitive decline in PD, possibly due to noradrenergic dysfunction (Sommerauer et al., 2018).

Based on the considerations described above, the aim of this ultra-high-field MRI study is to compare 7T MRI neuromelanin related signal intensity between PD patients and HC in both the SN and LC. We also aim to explore the association between the signal intensity in the SN and LC and cognitive performance in PD patients. While previous studies are hampered both by inadequate resolution and a potential bias stemming from manual segmentation. This is the first ultra-high field MRI study investigating NM related changes in a relatively large cohort of PD patients and HC. In addition, automated techniques for the determination of the SN and LC regions-of-interest are applied. The development of automated analysis techniques is important as it generates more objective results, facilitates easier replication of study methods, and enhances translatability to clinical practice.

## 2 Materials and methods

This study was conducted using baseline data from the TRACK-PD study, a longitudinal, observational 7T MRI study in PD patients. The study protocol, including details related to the MRI acquisition and neuropsychological assessment, was extensively described elsewhere (Wolters et al., 2020).

### 2.1 Participants

Early-stage PD patients and age and sex-matched HC participants were recruited from the Maastricht University Medical Hospital and the community between July 2019 and December 2021. All PD patients had to be diagnosed with idiopathic PD (according to the UK Parkinson’s Disease Brain Bank criteria) within the last three years before inclusion (Hughes et al., 1992). Participants with advanced cognitive impairment, defined as a score of <24 on the Montreal Cognitive Assessment (MoCA), a diagnosis of dementia according to the fifth edition of the Diagnostic and Statistical Manual of Mental Disorders (DSM 5 (Association AP, 2013) criteria or other neurodegenerative diseases, were excluded from participation. Potential participants were also excluded if they had any contra-indications for 7T MRI acquisition, such as claustrophobia or ferromagnetic implants. These exclusion criteria were also in place for the HC group.

Written informed consent was obtained from all participants prior to study participation. The study was approved by the local institutional review board (METC AZM/UM 18-027) and performed in accordance with the principles of the Declaration of Helsinki.

### 2.2 Clinical and neurological examination

Demographic and disease related characteristics were documented, including the levodopa equivalent daily dose (LEDD) (Tomlinson et al., 2010). Motor functions in PD patients were assessed with the Unified Parkinson’s Disease rating scale (MDS-UPDRS) (Goetz et al., 2008) and Hoehn and Yahr scale (Goetz et al., 2004), by certified clinicians. For PD patients, all tests were performed while in the medication “ON” state.

For the neuropsychological assessment a screening test battery was composed which assesses the most important cognitive domains. This test battery was also largely aligned with the neuropsychological test battery of other PD studies (Fereshtehnejad et al., 2017, Bloem et al., 2019), which enables potential future validation studies or data exchange. Overall cognitive functions were evaluated with the Montreal Cognitive Assessment (MoCA) (Nasreddine et al., 2005). This was followed by a neuropsychological assessment battery, consisting of the following standard tests: 1. The 'Phonemic and Semantic Fluency Test' for executive function (Gladsjo et al., 1999); 2. The 'Auditory Verbal Learning Test' for memory (15 Words Test) (Vakil and Blachstein, 1993); 3. The 'Benton Judgment of Line Orientation' for visuospatial function (Benton et al., 1978); 4. The 'Symbol Digit Modalities Test' (SDMT) for mental speed and attention (Smith, 1982); and 5. The 'Letter Number Sequencing Test' for working memory (Wechsler, 2008).

Previous studies suggest an association between degeneration of the LC and REM-sleep behaviour disorders (RBD) in PD (García-Lorenzo et al., 2013). For this reason we assessed RBD symptoms of all participants with the 'REM-sleep behaviour disorder screening questionnaire' (RBDSQ). This self-reporting questionnaire is a validated 13-item screening tool for the detection of REM sleep behaviour disorders (Stiasny-Kolster et al., 2007). A total score of five points or more is considered as a positive test result (Stiasny-Kolster et al., 2007).

### 2.3 MR imaging acquisition

Participants were scanned on a 7T MRI scanner (Magnetom, Siemens, Erlangen, Germany) equipped with a Nova Medical 32-channel head coil. Dielectric pads were applied to improve the signal in the temporal brain regions (Teeuwisse et al., 2012). In PD patients, the MRI was carried out while in the “ON” medication state to reduce motion artefacts due to tremor and to reflect clinical practice.

The 7T protocol included a localizer sequence, which was acquired for optimal planning. B0 and B1 mapping and shimming were performed to correct for field inhomogeneities. Secondly, a whole-brain MP2RAGE (Magnetization Prepared 2 Rapid Acquisition Gradient Echoes) scan was performed, with the following parameters: Repetition Time (TR) 5000 ms; Echo Time (TE) 2.51 ms; flip angle (FA) 5 and 3°; Field-of-view (FoV) 208×208 mm^2^; Voxel size 0.65×0.65×0.65 mm^3^; 240 sagittal slices; resulting in an anatomical T1-weighted image. This MP2RAGE was combined with a sagittal SA2RAGE (Saturation Prepared with 2 Rapid Gradient Echoes) scan: TR/TE, 2400/0.78 ms; FA 4 and 10°; FoV 256×256 mm^2^; Voxel size 2.0×2.0×2.0 mm^3;^ 88 sagittal slices. This scan was used to eliminate any B1-related biases (Eggenschwiler et al., 2012). Furthermore, a magnetization transfer-weighted turbo flash (MT-TFL) (Priovoulos et al., 2018) was performed, with 3 cm coverage of the brainstem region, used to visualise the SN and LC. The following parameters were used for this scan: TR/TE, 538/4.08 ms; FA 8°; FoV 192×192 mm^2^; Voxel size 0.4×0.4×0.5 mm^3;^ 60 axial slices (Priovoulos et al., 2018). The orientation of the MT-TFL slices was set perpendicular to the brainstem and it covered the part between the upper border of the mesencephalon and the lateral recess of the 4th ventricle (Wolters et al., 2020).

### 2.4 MRI quality control and pre-processing

First, raw DICOM images were converted to nifti format. Then, MRI quality control was performed by visual inspection of all images. Participants with distorted brain images, including ghosting, motion or pulsation artefacts affecting the SN or LC region were excluded from analysis. This also applied to participants of whom the SN or LC region was localised outside the FoV of the MT-TFL images. In total, 150 participants provided MRI data. Eight participants were excluded because either the SN or LC region was located outside the FoV of the MT-TFL images. Furthermore, 28 participants were excluded because of motion or pulsation artefacts in the SN or LC region. In total, 27 PD and nine HC participants were excluded from analysis.

Structural T1-weighted images of the remaining participants were pre-processed using FreeSurfer (v6.0) software (https://surfer.nmr.mgh.harvard.edu/). This included the processing steps of non-uniform signal correction, signal and spatial normalizations, skull stripping and brain tissue segmentation.

MT-TFL images were skull stripped with the Brain Extraction Tool (BET) in FSL (v6.0) (Smith, 2002). Next, for all individual participants the MT-TFL images were linearly registered to the anatomical T1-weighted structural images using the boundary-based registration (BBR) in FreeSurfer and the FLIRT toolbox in FSL (Greve and Fischl, 2009, Jenkinson et al., 2002, Jenkinson and Smith, 2001). All registrations were visually checked and a manual correction was applied if necessary with ITK-SNAP 4.0 software (Yushkevich et al., 2006).

### 2.5 MRI analysis

**Fig. 1:**
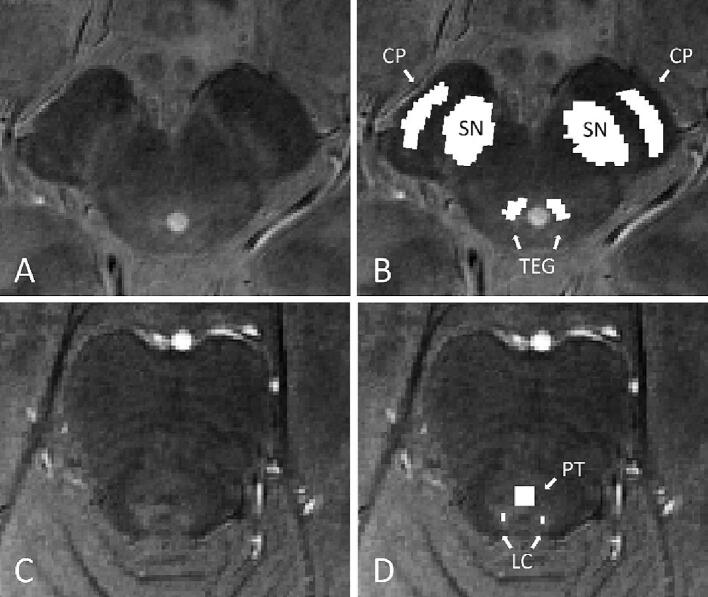
Visualisation of the region-of-interest and reference masks. (A) Axial MT-TFL brainstem slice at the level of the mesencephalon; (B) ROI masks in the bilateral substantia nigra (SN) region and reference masks in the bilateral cerebral peduncle (CP) and tegmentum (TEG). (C) Axial brain stem slice at the pontine level; (D) ROI masks in the bilateral locus coeruleus (LC) region and a reference mask in the pontine tegmentum (PT).

The probability maps of the ‘Atlas of the Basal Ganglia’ (ATAG) were used for the creation of the SN region-of-interest (ROI). This atlas has been created using ultra-high resolution 7T MRI data of 30 young, 14 middle-aged and 10 elderly participants (Keuken et al., 2017). For this study, the elderly atlas of the SN probability maps was used. Within these maps a separate mask of the left and right SN was drawn at the lowest level on which the SN could be well visualised. These masks were then converted to a voxel size of 0.65×0.65×0.65 mm^3^ and registered to the anatomical T1-weighted images of the individual participants through a linear transformation with the FLIRT toolbox in FSL (Jenkinson et al., 2002, Jenkinson and Smith, 2001). All masks were visually checked and if necessary minor manual corrections were applied with FSLeyes nudge. Corrected masks were then resampled with FSL FLIRT. Two reference masks (left and right) were drawn at the same level as the SN ROI in the cerebral peduncle (CP) and tegmentum (TEG). These reference masks were converted to a 0.65×0.65×0.65 mm^3^ voxel size and registered to the anatomical T1-weighted images alike the SN ROI (Fig. 1).

Because of the small size of the LC it was chosen not to manipulate the MT-TFL sequence and to, unlike the SN analysis, directly use these images as the target for the LC analysis. In this manner, interpolation artefacts were avoided while preserving the original signal (Guinea-Izquierdo et al., 2021). The raw MT-TFL data were used to create a template for the whole dataset utilizing “buildtemplateparallel”, which is part of the Advanced Normalization Tools (ANTs). Within this template a mask was drawn manually for both the left and right LC. Next, within the centre of this LC mask, a 1×2 voxel mask (∼0.4 × 0.8 mm^2^) centred over the highest intensity voxels of three consecutive slices, was defined as the LC ROI. At the same level a reference mask was drawn in the pontine tegmentum (PT), equidistant from both LC masks and with the apex of the 4th ventricle as a dorsal limit, with a total size of 2.4×2.4×1.5 mm^3^ (Fig. 1).

Mean signal intensity of the SN and LC was calculated and normalized to the mean signal intensity values of the surrounding reference ROIs (CP and TEG for the SN and PT for the LC). The contrast-to-noise ratio (CNR) of the SN and LC signal intensity was calculated using the following formula:

CNR=(SIroi-SIref)/SIref

Where *SIroi* is the signal intensity measured in the SN or LC ROI and *SIref* is the reference signal intensity in respectively the cerebral peduncle and tegmentum region or pontine tegmentum.

### 2.6 Statistical analysis

Data were statistically analysed using IBM SPSS version 25 (IBM Corp. Released 2017. IBM SPSS Statistics for Windows, Version 25.0. Armonk, NY: IBM Corp). Demographic and disease-related variables between PD and HC were compared with Pearson’s chi-squared test for categorical variables (non-parametric test), the Mann-Whitney *U* Test for ordinal variables and non-normally distributed continuous variables and the student’s *t*-test for normally distributed continuous variables. The Shapiro-Wilk test was used to assess the normality of the data. The statistical significance threshold was set at p < 0.05.

Potential differences in CNR ratios of the SN and LC between PD and HC participants were assessed with the student’s *t*-test for normally distributed variables and the Mann-Whitney *U* Test for non-normally distribute variables. In addition, since age is known to influence signal intensity on neuromelanin-sensitive MRI sequences (Shibata et al., 2006), an analysis of covariance (ANCOVA) was performed including age as a covariate. A p-value < 0.05 was considered to be statistically significant.

Correlation analysis was performed using Spearman’s correlation. For the whole group of participants, correlations were investigated between CNR values of the SN and LC and cognitive test scores and age. For the PD participants specifically, correlations were assessed between the SN and LC CNR values and both the total MDS-UPDRS III motor score and right- and left lateralized MDS-UPDRS III motor scores. The lateralized MDS-UPDRS III scores were calculated by adding together the right- and left-sided items of tremor, rigidity and bradykinesia (items 20–25 and 32–34) (Goetz et al., 2008). Bonferroni correction was applied to correct for multiple comparisons, resulting in a significance value of p < 0.004 for the correlation analyses.

Data availability Data of the TRACK-PD study cohort is available for other investigators on request. A formal data sharing agreement and approval of the local ethics committee is mandatory.

## 3 Results

### 3.1 Demographic and clinical characteristics

A group of 78 early-stage PD patients and 36 HC participants were included. As presented in Table 1, no significant differences in demographic characteristics were found between PD patients and HC. Although no significant difference was found between PD and HC regarding the overall cognitive performance assessed with the MoCA test (p = 0.583), PD participants performed worse on the SDMT (p = 0.001), which evaluated mental speed and attention. None of the other cognitive tests showed a significant difference between the two groups. To correct for age, sex and education, Z-scores were calculated for each cognitive test using Dutch normative values (Supplementary data, Table 1). In addition, RBDSQ scores of the PD participants did not significantly differ from the HC group (p = 0.087). However, when a RBDSQ test score of five or higher is regarded as positive, it can be concluded that 32 (41.0%) of the PD patients have RBD, whereas 8 (22.2%) of the HC subjects demonstrate the same.

**Table 1:** Demographic and clinical features.

	PD (n = 78)	HC (n = 36)	*P*-value
Sex (n (%) male)	57 (73.1)	25 (69.4)	0.688
Age (years)	62.6 (7.9)	61.1 (8.6)	0.431
Handedness (n (%) right)	68 (87.2)	33 (92.7)	0.484

Level of education completed (n (%))			0.556
Primary	0.0 (0.0)	0.0 (0.0)	
Secondary	18 (23.1)	10 (27.7)	
Lower tertiary	44 (56.4)	14 (38.9)	
Higher tertiary	16 (20.5)	12 (33.3)	
Time since diagnosis (months)	17.6 (8.9)		
MDS UPDRS III score	19.0 (8.0)		
Hoehn & Yahr stage (1/2/3/4/5)	27/48/3/0/0		
Side of onset (left/right/unknown)	41/34/3		
LEDD (mg/day)	370.0 (206.4)		
RBDSQ	3.4 (4.1)	1.9 (3.3)	0.087

MoCA	27.9 (1.6)	27.8 (1.6)	0.583
Phonemic fluency	39.7 (11.1)	38.5 (8.6)	0.587
Semantic fluency	39.1 (8.7)	40.5 (7.1)	0.294

15 Words Test			
Total score (0–75)	37.3 (10.0)	39.1 (9.3)	0.387
Maximum immediate recall (0–15)	10.2 (2.5)	10.5 (2.3)	0.519
Retention score (MRET1PC) (%)	92.6 (30.8)	106.1 (52.8)	0.161
Retention score (MRET2PC) (%)	71.8 (21.9)	77.3 (20.7)	0.122
BJOL	26.9 (3.3)	26.9 (3.0)	0.900
LNST	18.8 (2.3)	18.9 (2.2)	0.517
SDMT	44.9 (10.5)	52.8 (11.7)	0.001*

MDS UPDRS III = Movement Disorders Society sponsored revision of the Unified Parkinson’s disease Rating Scale-Part III (severity of motor symptoms); LEDD = Levodopa Equivalent Daily Dose; RBDSQ = REM-sleep behaviour disorder screening questionnaire; MoCA = Montreal Cognitive Assessment; BJOL = Benton Judgment of Line Orientation; LNST = Letter Number Sequencing Test; SDMT = Symbol Digit Modalities Test.

Results are represented as mean (SD), unless otherwise specified.

### 3.2 Signal intensity analysis in the LC and SN

**Fig. 2:**
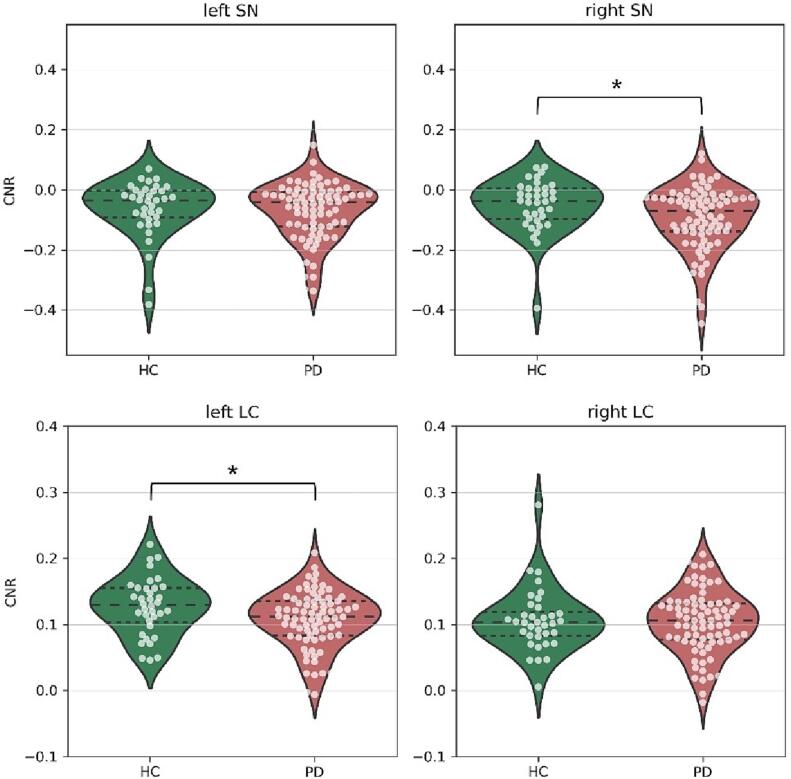
Comparison of SN and LC CNR values between PD and HC groups. Dashed lines represent the quartiles of the distribution. A significant difference in CNR values between PD and HC was found in the right SN (HC: mean = −0.049, std = 0.082, PD: mean = −0.090, std = 0.102; p = 0.029) and left LC (HC: mean = 0.127, std = 0.043, PD: mean = 0.108, std = 0.043; p = 0.027).

Compared with HC, PD participants showed significantly lower CNR values in the right SN region (HC: mean = −0.049, std = 0.082, PD: mean = −0.090, std = 0.102; p = 0.029) and left LC (HC: mean = 0.127, std = 0.043, PD: mean = 0.108, std = 0.043; p = 0.027). No differences in CNR values were found between PD and HC participants in the left SN region (HC: mean = −0.051, std = 0.069, PD: mean = −0.066, std = 0.089; p = 0.446) and right LC (HC: mean = 0.106, std = 0.048, PD: mean = 0.104, std = 0.048; p = 0.826) (Fig. 2). Also, no group differences were found when averaging the left and right SN (HC: mean = −0.058, std = 0.087, PD: mean = −0.084, std = 0.094; p = 0.109) or the left and right LC (HC: mean = 0.117, std = 0.041, PD: mean = 0.106, std = 0.038; p = 0.165).

The ANCOVA, correcting for age effects, showed a significant different CNR value between PD and HC in the left LC (p = 0.017). However, by adding age as a confounder, the CNR values of the right SN did not differ anymore between PD and HC (p = 0.055).

### 3.3 Correlation analysis

**Fig. 3:**
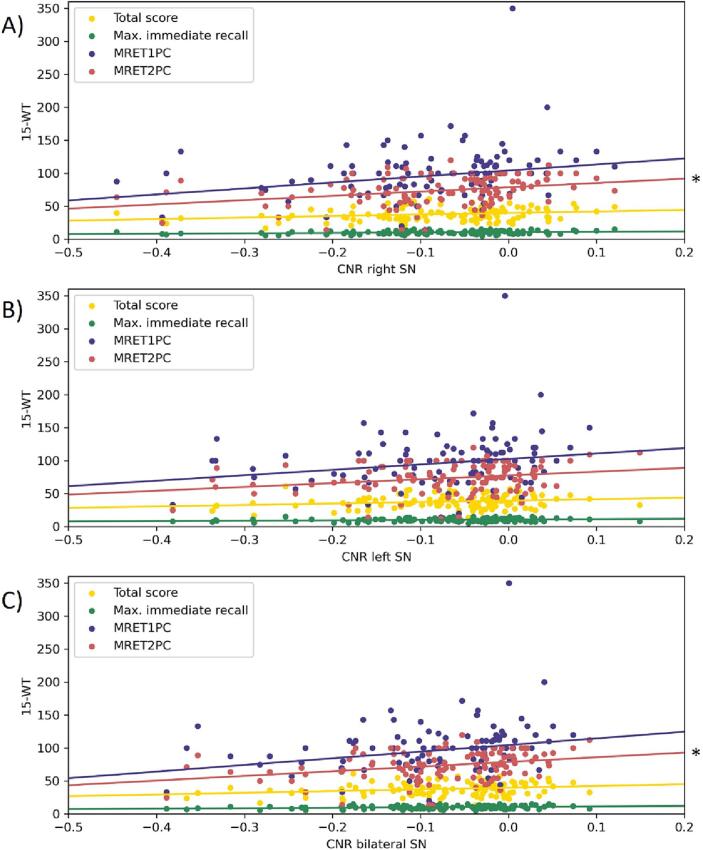
Correlation between SN CNR values and 15 words test scores for the whole group of participants. (A) Correlation between the CNR of the right SN and 15 words test scores. There was a significant positive correlation between the right SN CNR and the MRET2PC score (p = 0.001, r_s_ = 0.297). (B) Correlation between the CNR of the left SN and 15 words test scores. No significant correlations were found for the left SN CNR. (C) Correlation between mean CNR of the bilateral SN and 15 words test scores. There was a significant positive correlation between the CNR of the bilateral SN and the MRET2PC score (p = 0.002, r_s_ = 0.287).

For the PD and HC participants combined, a positive correlation was found between the CNR of the right SN and the second 15- wt retention score (MRET2PC, p = 0.001, *r_s_* = 0.297). There was also a trend towards a positive correlation between the CNR of the right SN and the first 15- wt retention score (MRET1PC, p = 0.007, *r_s_* = 0.251) and the MoCA total score (p = 0.009, *r_s_* = 0.243). No significant correlation was found between the CNR of the left SN and any of the cognitive scores. In addition, a positive correlation between the mean CNR of the bilateral SN and the second 15- wt retention score (MRET2PC, p = 0.002, *r_s_* = 0.287) was found. There was also a trend towards a positive correlation between the mean CNR of the bilateral SN and the first 15- wt retention score (MRET1PC, p = 0.004, *r_s_* = 0.265) (Fig. 3). Both retention scores are assessing delayed recall. No statistically different correlation was found between the CNR of the LC and any of the cognitive outcome measures.

In addition, both groups were separately compared regarding associations with motor and cognitive scores. For the HC group, no significant correlations were found between the CNR of the SN or LC and any of the cognitive measures. For the PD group, a positive correlation was found between the CNR of the right SN and the maximum immediate recall score of the 15- wt (p = 0.003, *r_s_* = 0.336) and a trend for a positive correlation between the CNR of the right SN and the 15- wt total score (p = 0.009, *r_s_* = 0.295*)* (Supplementary material, Figs. 1 and 2).

Another correlation analysis was performed assessing the association between LC and SN CNR and lateralized MDS-UPDRS III motor scores. While there seemed to be a positive correlation between the right LC CNR and right-lateralized motor score and a negative correlation between the right LC CNR and left-lateralized motor scores visually, this relationship was not found to be statistically significant (p = 0.014, *r_s_* = 0.277 and p = 0.016, *r_s_* = −0,273, respectively). Also the left LC CNR and left and right SN CNR did not show a significant correlation with the lateralized motor scores (Supplementary material, Fig. 3).

## 4 Discussion

In this ultra-high-field 7T MRI study neuromelanin related signal intensity of the SN and LC was compared between a large group of early-stage PD patients and controls. PD participants showed lower CNR values in the right SN and left LC. However, the finding in the SN did not survive age-correction. Upon examining the associations with cognitive scores, we found that lower CNR in the SN was associated with worse memory performance in the whole group as well as in the PD patients separately.

The SN and LC modulate various cognitive and behavioural functions and have a proposed role in the pathophysiology of several neurodegenerative diseases. For this reason, they make an interesting target for studies searching for imaging biomarkers. In addition to PD, lower signals on neuromelanin-sensitive MRI sequences in these nuclei have been reported in several other neuropsychiatric diseases, such as Alzheimer’s disease or depressive disorders (Liu et al., 2017). Until now, only two 7T MRI studies have been published comparing neuromelanin related signal changes in the LC between PD and HC. Both found a lower signal intensity in the LC of PD patients compared to HC, but only in the caudal portion. There were no differences between PD and HC when assessing the LC as a whole (O’Callaghan et al., 2021, Ye et al., 2022). Despite being not able to visualize the caudal part of the LC with our current MRI protocol, we were able to demonstrate that alterations between early-stage PD and HC can also be found in the central portion of the LC.

Lateralized results were also reported by one of the previous 7T MRI studies, with signal changes in PD patients found in the right, but not in the left, caudal LC (Ye et al., 2022). The exact role of the LC in motor control and its association with ipsi- and contralateral motor symptoms is not fully clarified. Previous studies have yielded contrasting results. Some report an association between LC integrity and ipsilateral motor symptoms (Ye et al., 2022), while others have observed a more significant decrease in LC signal intensity contralateral to the clinically most affected side (Doppler et al., 2021). In the current analysis we searched for a correlation between LC CNR and lateralized motor scores. However, no significant correlation was found between the LC CNR and lateralized motor symptoms (Supplementary data, Fig. 3). The exact relation between LC integrity and motor symptoms therefore remains unclear.

This is the first 7T MRI study comparing neuromelanin related signal changes in the SN between PD and HC. However, no differences were demonstrated between groups in this region. These results imply that the LC is affected more early in the disease course compared to the SN, which is in line with the bottom-up progression of disease theory based on the pathological studies by Braak and colleagues (Braak et al., 2002). Although this theory seems to be applicable to a significant proportion of PD patients, recent evidence suggest that some PD cases display an alternative route of disease progression, starting in the olfactory bulb. In this group of patients PD pathology mainly affects the amygdala region, with limited or no brainstem involvement (Borghammer et al., 2022).

In contrast, various 3T MRI studies have been performed assessing neuromelanin-sensitive signal intensity in the SN or LC. Several studies have reported a decrease in neuromelanin related signal intensity in PD patients compared to HC (Gaurav et al., 2021, Wang et al., 2021, Wang et al., 2018). Previous research comparing 3T and 7T MRI modalities determined that 7T MRI is superior in terms of localizing small brain structures (Isaacs et al., 2020). It is therefore remarkable that we did not find signal changes in the SN, while previous 3T MRI studies did report differences in PD patients in this region. A possible explanation for this could be related to Specific Absorption Rate (SAR) restrictions at 7T MRI. While SNR greatly increases with 7T MRI, the SAR restrictions cause the CNR of the MT-TFL sequence to be slightly decreased compared to 3T MRI (Priovoulos et al., 2018). It might therefore be harder to demonstrate group differences. However, since registration is critical when assessing small brain stem nuclei, the increased spatial resolution and contrast of 7T MRI allow for a more reliable localization and more valid analysis compared to 3T MRI.

We found a positive correlation between the SN CNR and performance on the 15 words test in PD patients. None of the other cognitive test scores displayed a correlation with either LC or SN integrity. Although we believe that the inclusion of an early-stage PD group, which did not show evident signs of cognitive impairment in comparison to the age-matched HC population, can explain this lack of correlation, it is still striking that a correlation was found with the 15- wt For the PD group this mainly concerned immediate recall and not delayed recall function. This corresponds with the believe that memory disorders in PD are related to retrieval rather than to consolidation failure (Costa et al., 2014). Liu et al. reported a similar result as they found a relationship between SN integrity and working memory deficits in PD (Liu et al., 2021). In addition, another paper reported an association between iron concentration in the SN and working memory in HC (Xu et al., 2021). It could therefore be possible that SN integrity is associated with memory function. However, studies on the involvement of the basal ganglia in cognitive functioning suggest a central role in learning, rather than it being associated with deficits in specific cognitive domains (Foerde and Shohamy, 2011). Besides assessing memory function, the 15- wt evaluates an individual's learning abilities, as they are presented with the same words in five repeated trials. It could therefore be hypothesized that the loss of SN integrity is not necessarily associated with impaired memory function but with an impaired learning function in general, which could explain why only a correlation with this specific neuropsychological test is found. The correlation analysis in the current study is exploratory and results should be interpreted with caution. To further establish the relationship between the SN integrity, memory function and learning capabilities, future longitudinal studies are necessary.

Results in this study were corrected for age, since this is known to influence signal intensity on neuromelanin-sensitive MRI sequences (Shibata et al., 2006). In addition to age, previous literature suggests a correlation between several clinical symptoms and degeneration of the locus coeruleus. This includes cognitive impairment, RBD and psychiatric complaints (Li et al., 2019, Ye et al., 2022, Prasuhn et al., 2021, Del Tredici and Braak, 2013, Nobileau et al., 2023). The RBD questionnaire included in this study, did not significantly differ between PD and HC. As for the cognitive evaluation, only the results of the SDMT differed significantly between PD and HC. Other cognitive tests did not show a difference between groups. Also, no correlation was found between the individual cognitive test scores and the neuromelanin related signal intensity in the LC. We believe that this might be caused by the fact that an early-stage PD group was included in this study, which did not show evident signs of cognitive impairment in comparison to the age-matched HC population. The study of Li et al. also showed that altered CNR values in the LC are less apparent in early PD without cognitive impairment compared to PD with cognitive complains (Li et al., 2019). It would therefore be interesting to repeat the current analysis on longitudinal data, too see whether the development of cognitive impairment over time is correlated with a decrease of neuromelanin related signal intensity in the LC. However, for this current analysis we believe that neither RBD symptoms nor cognitive complains have had a confounding effect on the study results.

This study has several limitations. First of all, due to technical restrictions the FoV for the NM sensitive MRI sequence was relatively small. Since we aimed to examine signal intensity in both the SN and LC, we had to exclude several participants because either the SN or LC was located outside the FoV. Furthermore, since the cranial part of the SN and the caudal part of the LC was often positioned outside the FoV, volumetry measurements or spatial pattern analysis could not be performed. This is unfortunate since recent studies suggest a spatial pattern of LC and SN degeneration (Madelung et al., 2022, Biondetti et al., 2020). Future ultra-high field studies focussing on the spatial organisation of the LC and SN are therefore necessary. Another limitation relates to (involuntary) motion artefacts, as these are one of the biggest challenges in high-resolution imaging (Ladd et al., 2018). This is especially true for visualising parts of the brainstem, since there is a close relation to surrounding vascular structures and cerebrospinal fluid compartments. In this study several participants displayed artefacts in either the SN or LC and have therefore been excluded from analysis. The reasons described above resulted in a relatively large exclusion rate for this study. Future ultra-high-field MRI studies, specifically aiming to incorporate both the midbrain and the pontine catecholaminergic nuclei, should use a more extensive MRI protocol with a larger FoV, so that all relevant nuclei can be fully visualised.

In addition, this current study only consists of PD patients and HC. In order to investigate if NM related signal intensity in the LC can also differentiate PD from patients with other neurodegenerative diseases, such as atypical parkinsonism, future studies should include a control group with other neurodegenerative diseases. This would help to differentiate between PD related alterations and changes which occur due to aging or other neurodegenerative processes.

Lastly, this study focusses on neuromelanin related signal intensity, which is a very specific potential MRI biomarker. There probably is no single method with the potential to serve as the perfect PD biomarker and different biomarker techniques are likely to complement each other (Weingarten et al., 2015). However, we believe that in order to determine an ideal set of combined biomarkers for PD, it is important to first adequately study the individual markers and to develop optimal methods for analysis. In this way the most promising biomarkers can be determined, which can then be combined in future studies. Besides MRI, nuclear imaging techniques visualizing the noradrenergic and dopaminergic system, could also serve a valuable role in this. Eventually, a combination of different biochemical and imaging techniques is expected to have the best results for both the diagnosis and monitoring of PD.

In conclusion, the results of this study confirm that early-stage PD patients exhibit lower signal intensity in the LC compared to HC. No differences between groups were found in signal intensity of the bilateral SN. In addition, loss of SN integrity might influence working memory or learning capabilities in PD patients. Although 7T MRI yields advantages in terms of increased spatial resolution and better localisation of small brain stem nuclei, group comparisons might be influenced by a small decrease in CNR. Future studies should preferably investigate longitudinal signal changes in the LC and SN to further elucidate the pathophysiological mechanisms of PD.

## Declaration of Competing Interest

The authors declare that they have no known competing financial interests or personal relationships that could have appeared to influence the work reported in this paper.

## Data Availability

Data will be made available on request.
