# Supplementary material for: Neuromelanin related ultra-high field signal intensity of the locus coeruleus differs between Parkinson’s disease and controls
Source: Neuroimage Clin. 2023 Jul 22;39:103479. doi: 10.1016/j.nicl.2023.103479 (PMC10394012; doi:10.1016/j.nicl.2023.103479)
Supplement: Supplementary data 1 [file mmc1.docx]

| Table 1. Neuropsychological test scores (z-scores) | | | | |
| --- | --- | --- | --- | --- |
|  | | PD (n = 78) | HC (n = 36) | *P*-value |
| Phonemic fluency | | -0.01 (1.1) | -0.14 (0.8) | 0.465 |
| Semantic fluency | |  |  |  |
|  | Animals | -0.45 (0.8) | -0.25 (0.6) | 0.216 |
|  | Professions | -0.41 (0.8) | -0.35 (0.6) | 0.704 |
| 15 Words Test | |  |  |  |
| Total score | | -0.90 (1.0) | -0.73 (1.1) | 0.459 |
| Immediate recall | | -0.66 (1.2) | -0.40 (1.1) | 0.259 |
| Retention score | | -0.31 (1.2) | 0.01 (1.1) | 0.185 |
| BJOL | | 0.59 (0.7) | 0.61 (0.7) | 0.908 |
| LNST | | 0.16 (0.9) | 0.01 (1.1) | 0.786 |
| SDMT | | 0.46 (1.0) | 1.3 (1.2) | 0.001* |
| *BJOL = Benton Judgment of Line Orientation; LNST = Letter Number Sequencing Test; SDMT = Symbol Digit Modalities Test.*  Results are represented as mean z-scores (SD), unless otherwise specified. | | | | |


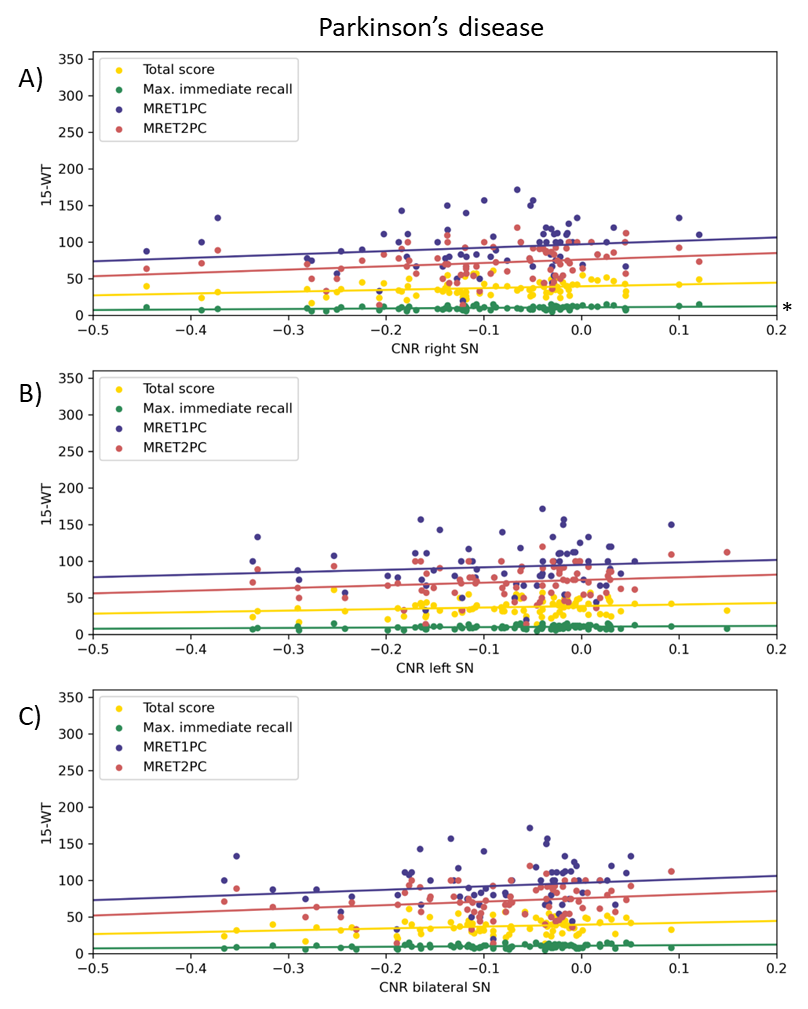


***Figure 1.*** *Correlation between SN CNR values and 15 words test scores for the PD group. (A) Correlation between right SN CNR and 15 words test scores. A positive correlation was found between the CNR of the right SN and the maximum immediate recall score (p = 0.003, rs = 0.336). (B) Correlation between the CNR of the left SN and 15 words test scores. No significant correlations were found for the left SN CNR. (C) Correlation between mean CNR of the bilateral SN and 15 words test scores. No significant correlations were found for the CNR of the bilateral SN.*


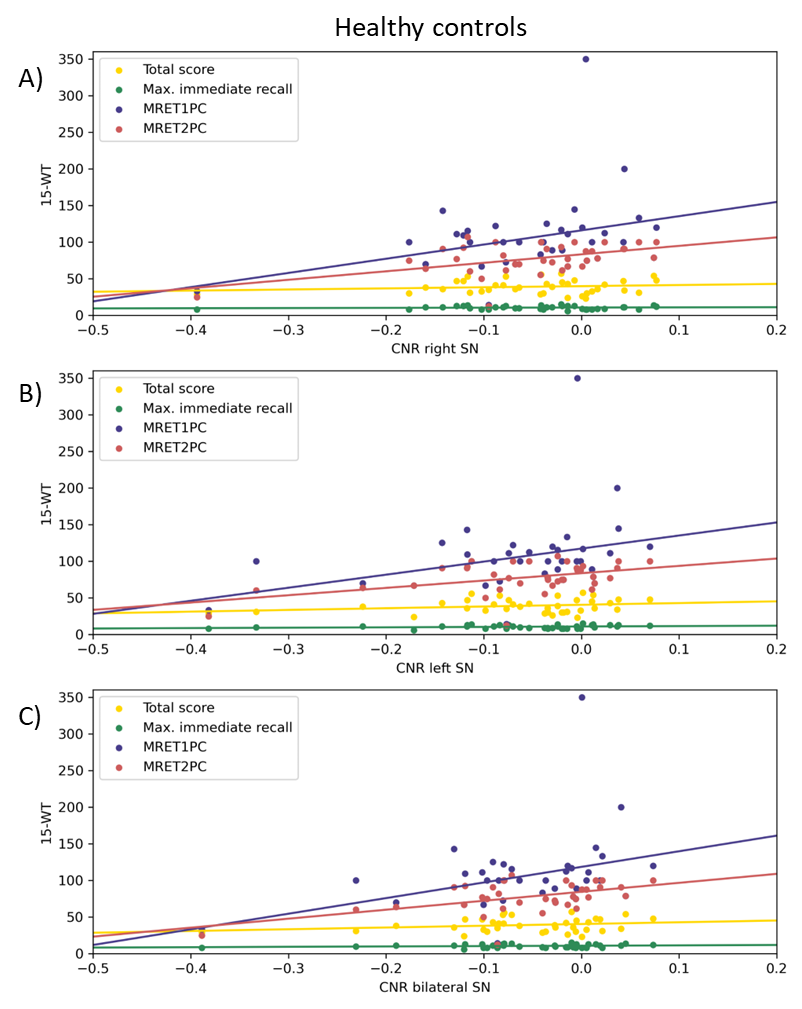


***Figure 2.*** *Correlation between SN CNR values and 15 words test scores for the HC group. (A) Correlation between the CNR of the right SN and 15 words test scores. No significant correlations were found for the right SN CNR. (B) Correlation between the CNR of the left SN and 15 words test scores. No significant correlations were found for the left SN CNR. (C) Correlation between mean CNR of the bilateral SN and 15 words test scores. No significant correlations were found for the bilateral SN CNR.*


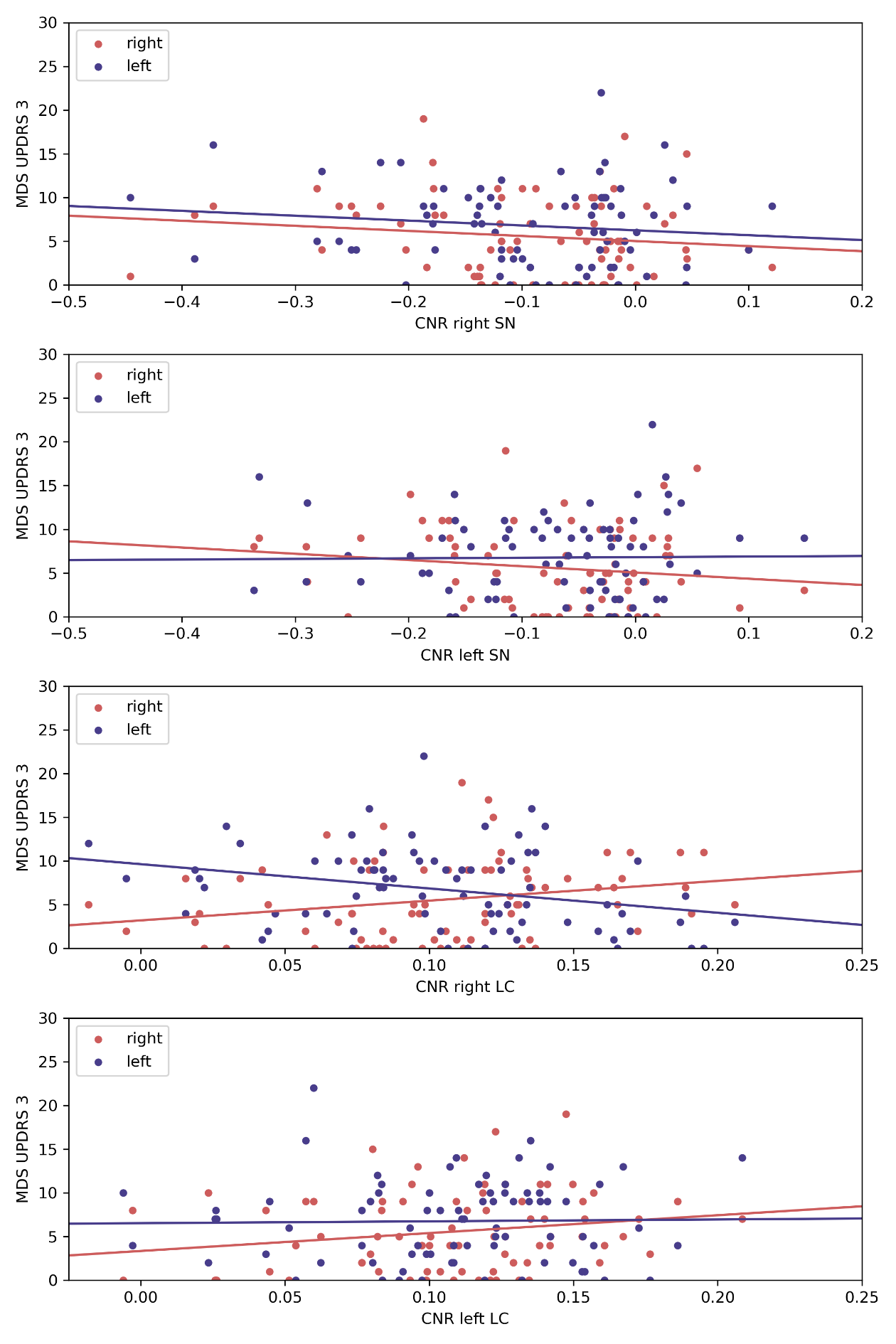


***Figure 3.*** *Correlation between LC and SN CNR values and right- and left-lateralized MDS-UPDRS III motor scores. No significant correlation was found between any of these measures.*
